# Uncoupling of the LKB1-AMPKα Energy Sensor Pathway by Growth Factors and Oncogenic BRAF^V600E^

**DOI:** 10.1371/journal.pone.0004771

**Published:** 2009-03-10

**Authors:** Rosaura Esteve-Puig, Francesc Canals, Núria Colomé, Glenn Merlino, Juan Ángel Recio

**Affiliations:** 1 Animal Models and Cancer Laboratory, Medical Oncology Research Program, Vall d'Hebron Research Institute-VHIO, Vall d'Hebron Hospital, Barcelona, Spain; 2 Proteomic Laboratory, Medical Oncology Research Program, Vall d'Hebron Research Institute-VHIO, Vall d'Hebron Hospital, Barcelona, Spain; 3 Laboratory of Cancer Biology and Genetics, National Cancer Institute, National Institutes of Health, Bethesda, Maryland, United States of America; Ordway Research Institute, United States of America

## Abstract

**Background:**

Understanding the biochemical mechanisms contributing to melanoma development and progression is critical for therapeutical intervention. LKB1 is a multi-task Ser/Thr kinase that phosphorylates AMPK controlling cell growth and apoptosis under metabolic stress conditions. Additionally, LKB1^Ser428^ becomes phosphorylated in a RAS-Erk1/2-p90^RSK^ pathway dependent manner. However, the connection between the RAS pathway and LKB1 is mostly unknown.

**Methodology/Principal Findings:**

Using the UV induced HGF transgenic mouse melanoma model to investigate the interplay among HGF signaling, RAS pathway and PI3K pathway in melanoma, we identified LKB1 as a protein directly modified by HGF induced signaling. A variety of molecular techniques and tissue culture revealed that LKB1^Ser428^ (Ser431 in the mouse) is constitutively phosphorylated in BRAF^V600E^ mutant melanoma cell lines and spontaneous mouse tumors with high RAS pathway activity. Interestingly, BRAF^V600E^ mutant melanoma cells showed a very limited response to metabolic stress mediated by the LKB1-AMPK-mTOR pathway. Here we show for the first time that RAS pathway activation including BRAF^V600E^ mutation promotes the uncoupling of AMPK from LKB1 by a mechanism that appears to be independent of LKB1^Ser428^ phosphorylation. Notably, the inhibition of the RAS pathway in BRAF^V600E^ mutant melanoma cells recovered the complex formation and rescued the LKB1-AMPKα metabolic stress-induced response, increasing apoptosis in cooperation with the pro-apoptotic proteins Bad and Bim, and the down-regulation of Mcl-1.

**Conclusions/Significance:**

These data demonstrate that growth factor treatment and in particular oncogenic BRAF^V600E^ induces the uncoupling of LKB1-AMPKα complexes providing at the same time a possible mechanism in cell proliferation that engages cell growth and cell division in response to mitogenic stimuli and resistance to low energy conditions in tumor cells. Importantly, this mechanism reveals a new level for therapeutical intervention particularly relevant in tumors harboring a deregulated RAS-Erk1/2 pathway.

## Introduction

Melanoma is the most lethal human skin cancer and its incidence is rapidly rising world-wide [1]. The development of effective therapeutics designed to target melanoma requires a comprehensive understanding of the underlying biochemical and genetic processes contributing to melanocytic neoplasic transformation and the subsequent progression to an advanced melanoma disease stage. Therefore, dissecting the aberrant signaling pathways that are critical to melanomagenesis and understanding the mechanisms by which these pathways interact with each other has become the recent focus of research directed at melanoma therapeutic intervention.

Dysfunctional receptor tyrosine kinase (RTK) signaling, in particular through the hepatocyte growth factor (HGF) tyrosine kinase receptor c-Met signaling pathway, is one important hallmark of melanoma. HGF signaling activates Ras-Erk1/2 and PI3K-AKT pathways, and Ras pathway activation has been shown to play a role in melanoma development and maintenance [2]. Notably, BRAF, a downstream activator in the RAS pathway is mutated in nearly 70% of human melanoma (BRAF^V600E^ activating mutation) while NRAS activating mutations occurs in 30% of melanomas (NRAS^Q61L^ activating mutation) [3]. In addition, support for PI3K-AKT pathway signaling dysfunction in melanomagenesis has been demonstrated by the documented loss of the tumor suppressor PTEN-containing chromosomal region in 5–20% of melanomas as well as the over expression of AKT3 in the advanced stages of this disease [4], [5]. Strikingly, however, single mutations within these two pathways are not sufficient to promote melanoma development suggesting that a complex interplay of these aberrant signalling pathways, under poorly understood circumstances, promote melanomagenesis [2].

We chose to investigate the potential interplay among the HGF RTK signaling, the RAS Ras-Erk1/2 and the PI3K-AKT pathways using the HGF transgenic mouse model in which HGF is over-expressed and which develops melanoma in response to neonatal ultraviolet (UV) radiation. This model is unique in that it develops melanocytic neoplasms in stages that are highly reminiscent of the human cutaneous malignant melanoma with respect to biological, genetic and etiologic criteria [6], [7].

To begin the analysis, we first searched for possible molecular candidates with potential to mediate the HGF complex signaling and identified the multitasking serine/threonine kinase, LKB1 [8] as one candidate. LKB1 is involved in cell cycle control [9], [10], cellular energy metabolism [11] and cell polarity [12]. The cellular localization and activity of LKB1 is controlled through its interaction with the STE20-related adaptor (STRAD) and the armadillo repeat-containing mouse protein 25 (Mo25); [13], [14]. These finding led to the discovery that LKB1 is the upstream kinase to AMP-activated protein kinase (AMPK) and is linked to mTOR through the AMPK-TSC1/TSC2 cascade [15], [16], [17]. LKB1 is phosphorylated on at least 8 residues, and evidence suggests that LKB1 auto-phosphorylates itself on at least four of these, whereas the other four are phosphorylated by upstream kinases [8]. Recent studies show that human LKB1^Ser428^ (the equivalent mouse residue is LKB^Ser431^) is phosphorylated in response to mitogenic signals including EGF, TPA, elevated levels of cAMP as well as by PKCζ [18], [19], [20], [21], [22], where the EGF-mediated phosphorylation of LKB1^Ser428^ is dependent on the activation of p90^RSK^. Although experiments conducted in G361 melanoma cells indicate that this residue is involved in LKB1-mediated cell growth inhibition [8], [23], and several other investigations implicate LKB1^Ser431^ residue in the activation of AMPK and BRK1/BRSK2 kinases (SAD-B/SAD-A) [21], [24], [25], a recent publication stating that LKB1 phosphorylation in the C-terminal is not required for regulation of AMPK BRSK1/2 and cell cycle arrest contradicts the previous findings [26].

Importantly, sporadic mutations in the *lkb1* gene have been documented in cancers of the breast, pancreas, lung, prostate, cervical and ovary as well as in Peutz-Jeghers syndrome, a rare disorder characterized by the appearance of intestinal polyps and mucocutaneous melanocytic macules [27], [28], [29], [30], [31]. In Peutz-Jegher patients, LKB1 may function as a tumor suppressor and is associated with loss of heterozygosity or somatic mutation at the *lkb1* locus (for review [8]). More importantly, *lkb1* mutations have been described in melanoma [32] and based on this information we determined if LKB1 could function as a potential link between an activated RAS pathway and dysfunctional c-Met signaling and play a role in melanoma development and progression.

In this study, we identify the mouse LKB1^Ser431^ residue as a phosphorylation target, not only for EGF, but also for HGF signaling and demonstrate that this LKB1 phosphorylation is executed in an Erk1/2-p90^Rsk^-dependent manner, as previously described in response to EGF stimulation [18], [19]. We demonstrate that LKB1^Ser428^ residue is constitutively phosphorylated in cells harboring BRAF^V600E^ activating mutations, and is found frequently phosphorylated in mouse tumor samples with an increased receptor tyrosine kinase activity suggesting a functional connection between BRAF oncogenic pathway and LKB1. Interestingly, BRAF^V600E^ mutant cells show a very limited response to metabolic stress that appears to be mediated by mechanism that involves the uncoupling of the energy stress sensor pathway LKB1-AMPK-mTOR. Importantly, inhibition of RAS-Erk1/2 pathway in BRAF^V600E^ mutant melanoma cell lines restores the LKB1-AMPK-mTOR pathway response to metabolic stress promoting apoptosis in coordination with the BH3-family proteins Bad and Bim and the Bcl-2 family member Mcl-1.

## Results

### HGF induces LKB1^Ser428^ phosphorylation in a RAS-Erk1/2-p90^RSK^ pathway-dependent manner

**Figure 1:**
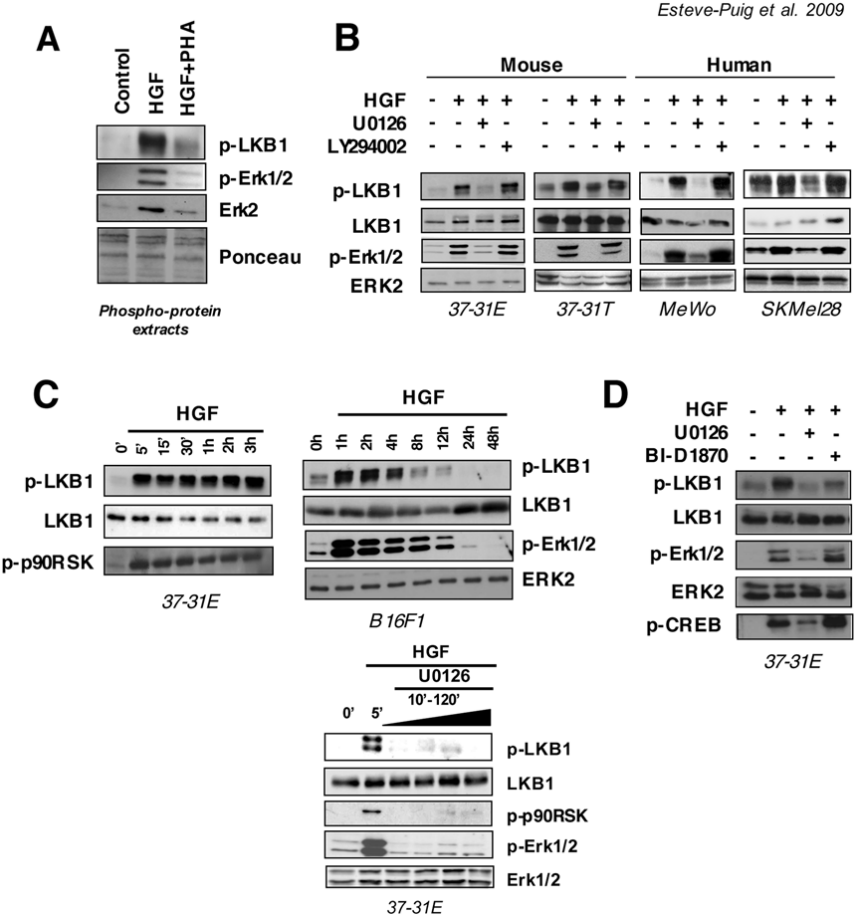
HGF induces LKB1^Ser431^ phosphorylation in a RAS-p90^RSK^ dependent manner. (A) Five µg of phospho-protein isolated complexes from samples: untreated (Control), HGF triggered (40 ng/ml) w/o PHA (0,2 µM) were resolved by SDS-PAGE. p-LKB1^Ser428^, p-Erk1/2^Thr202/Tyr204^ and Erk2 antibodies were probed against the membrane. Ponceau S staining of membrane is showed for phospho-protein extracts loading control. (B) 37-31E, 37-31T, SKMel28, and MeWo cells were treated in serum starvation conditions with HGF (40 ng/ml), U0126 (10 µM) and LY294002 (10 µM) as indicated in the figure. Western-blots show the levels of the indicated proteins. (C) Time course showing the phosphorylation of the LKB1^Ser431^ and p-90^RSK^ ^Thr359/Ser363^ after HGF triggering (40 ng/ml) under serum starvation conditions. LKB1 total protein is shown as a loading control. On the right, LKB1^Ser431^ is phosphorylated in response to HGF in an Erk1/2-p90^RSK^ dependent manner. Time course shows the phosphorylation of Erk1/2^Thr202/Tyr204^ and LKB1^Ser428^ in B16F1 cells. Down below, 37-31E melanoma cells were serum starved and triggered with HGF (40 ng/ml) for 5 minutes. Then, cells were treated with the Mek1/2 specific inhibitor U0126 (10 µM) for the indicated increasing times. Fifty µg of total lysates were resolved by SDS-PAGE and membrane was probed with the indicated antibodies. (D) 37-31E cells were treated for 10 min in serum starvation with HGF (40 ng/ml) in the presence or absence of U0126 (10 µM) or BI-D1870 (10 µM). Western-blots show the levels of p-LKB1^Ser431^, p-Erk1/2^Thr202/Tyr204^ and p-CREB^Ser133^.

We proposed to identify novel molecules involved in melanoma development and progression analyzing the HGF specific signaling in the UV induced HGF transgenic melanoma mouse model. To understand the HGF specific signaling contributions we used 37-31E-mouse melanoma cell line isolated from neoplasic lesions raised in the HGF transgenic-UV irradiated mice and performed a proteomic screening of the phospho-protein complexes induced after the growth factor treatment (data not shown). As a result, we identified LKB1 as a kinase that becomes phosphorylated in response to HGF. Since previous studies implicates RAS pathway in the modification of this residue we decided to use different cell lines harboring either, BRAF wild type (37-31E, 37-31T, B16F1 and MeWo) or BRAF^V600E^ mutant cell lines (UACC903, A375 and SKMel28). As shown in Figure 1A, in isolated phospho-protein complexes from 37-31E cells, LKB1^Ser431^ was specifically phosphorylated in response to HGF treatment since this phosphorylation was totally prevented by the pretreatment with the specific c-Met inhibitor PHA. LKB1^Ser428^ (Ser431 in mouse) is phosphorylated in response to EGF through RAS-Erk1/2-p90^RSK^ pathway [18], [20]. Since HGF triggering activates RAS and PI3K pathway [33], [34], [35], we used specific Mek1/2 (U0126) and PI3K (LY294002) inhibitors to determine which pathway was involved in the LKB1^Ser431^ phosphorylation. Figure 1B shows that in mouse and human melanoma cells, HGF-induced phosphorylation of LKB1^Ser431^ was totally abolished by the specific Mek1/2 inhibitor U0126, whereas the PI3K inhibitor LY294002 had no effect on HGF-induced phosphorylation of LKB1^Ser431^. Furthermore, time course experiments showed that p90^RSK^ became phosphorylated in response to HGF and its phosphorylation profile correlated with LKB1^Ser431^ phosphorylation (Fig. 1C). Additionally, analysis of B16F1 melanoma cells showed that the phosphorylation of the LKB1^Ser431^ was p-Erk1/2 dependent (Fig. 1C) and, inhibition of Mek1/2 after HGF treatment in 37-31E cells totally abolished the phosphorylation of Erk1/2, p90^RSK^ and LKB1^Ser431^ (Fig. 1C). To confirm the p90^RSK^ participation we used the p90^RSK^ specific inhibitor BI-D1870. Treatment of 37-31E cells with BI-D1870 abolished the HGF-mediated phosphorylation of LKB1^Ser431^ (Fig. 1D). The observed activation upon BI-D1870 treatment of Erk1/2 and the increased levels of p-CREB^Ser133^ is in agreement with the suggested p90^RSK^ negative-feedback loop that regulates Erk1/2 described by other authors [20].

These results show that, LKB1^Ser428^ is phosphorylated in response to HGF treatment in an Erk1/2-p90^RSK^dependent manner.

### LKB1^Ser428^ is phosphorylated in response to different growth factors and is highly phosphorylated in melanoma cells harboring BRAF^V600E^ mutations and in tumor samples

**Figure 2:**
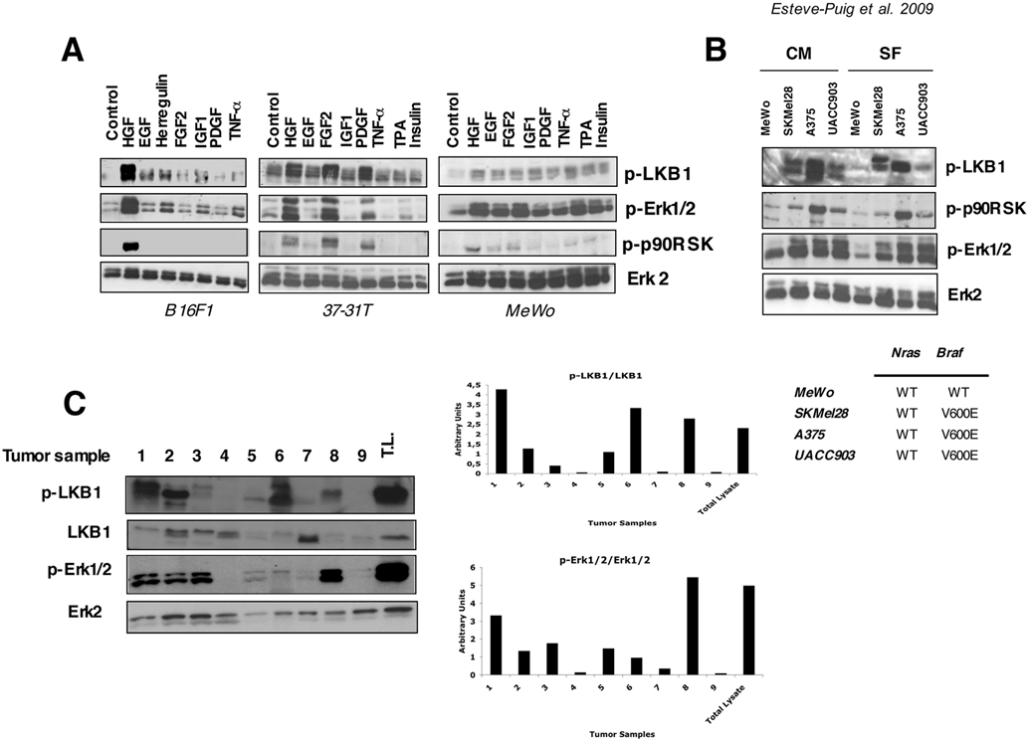
LKB1^Ser431^ (Ser428 human) is phosphorylated in response to different growth factors, in BRAF^V600E^ mutant melanoma cells and mouse tumor samples. (A) B16F1, 37-31T and MeWo cells were serum starved and treated with HGF (40 ng/ml), EGF (100 ng/ml), FGF2 (100 ng/ml), Herregulin (50 ng/ml), IGF-1 (50 ng/ml), PDGF (50 ng/ml), TNF-α (100 ng/ml) Insulin (100 nM) and TPA (200 nM). Fifty µg of total lysates were separated by SDS-PAGE and same membranes were incubated against the indicated antibodies. (B) MeWo (BRAF wild type), A375 (BRAF^V600E^), SKMel28 (BRAF^V600E^) and UACC903 (BRAF^V600E^) human melanoma cells were growth in complete medium (CM) or serum starvation (SF) conditions as indicated. Fifty µg of total lysates were analyzed by SDS-PAGE. The phosphorylation status of LKB1^Ser428^, p-Erk1/2^Thr202/Tyr204^ and p-90^RSK Thr359/Ser363^ is shown. Total Erk1/2 is used as a loading control. Cell genotypes are showed. (C) p-LKB1^Ser431^ and p-Erk1/2^Thr202/Tyr204^ levels in mouse melanoma tumor samples. Samples 1–7 primary tumors raised in HGF-UV irradiated transgenic mice. Samples 8 and 9 show xenograph tumors generated from 37-31E cells in FVB mice with high and low p-Erk1/2 levels, respectively. As a control fifty micrograms of protein from 37-31E melanoma cell line treated with HGF (40 ng/ml) for 10 minutes was added (Total lysates, T.L.). Same membrane was blotted against the indicated antibodies. Quantifications of phospho-proteins normalized against total protein are showed in the graphs below.

Next, we investigated if this post-translational modification was broader in scope. We tested several growth factors related to cancer development in human and mouse melanoma cell lines to check whether or not these ligands were able to induce LKB1^Ser428^ phosphorylation. As suggested by previous investigations [18], all ligands tested, including HGF, EGF, basic fibroblast growth factor (FGF2), insulin like growth factor 1 (IGF-1), platelet derived growth factor (PDGF), tumor necrosis factor-alpha (TNF-α), herregulin, insulin and phorbol-ester tumor promoter TPA were able to induced LKB1^Ser428^ phosphorylation in a cell type dependent manner (Fig. 2A). In all cases, the ligands that activated Erk1/2 and p90^RSK^ kinases led to the phosphorylation of LKB1^Ser428^ (Fig. 2A).

The aberrant regulation of the Ras-Erk1/2 pathway represents one of the hallmarks in cancer. Considering that 70% of human melanomas harbor BRAF activating mutations [3], we determined the phosphorylation status of LKB1^Ser428^ in different human melanoma cell lines harboring BRAF^V600E^ activating mutations in serum free and complete medium conditions. As expected, SKMel28, A375, and UACC903 human melanoma cell lines harboring the BRAF^V600E^ mutation demonstrated a constitutively phosphorylated LKB1^Ser428^ residue whereas the MeWo human melanoma cell line that harbors the wild type alleles did not (Fig. 2B).

Based on the above results, tumor samples with a deregulated tyrosine kinase pathway and/or with enhanced RAS-mediated mitogenic activity would be expected to exhibit elevated LKB1^Ser428^ phosphorylation. In this matter, the phosphorylation state of LKB1^Ser431^ in spontaneous tumor samples raised in UV-irradiated HGF transgenic mice and in xenographed tumors from the 37-31E-melanoma cells correlated with elevated levels of p-Erk1/2 (Fig. 2C).

All together, these data indicated the existence of a RAS pathway and LKB1 crosstalk suggesting that LKB1 might be involved in some of the RAS-Erk1/2 induced-responses, and more importantly would be contributing to BRAF oncogenic signaling.

### BRAF mutant melanoma cells have a dysfunctional LKB1-AMPK energy stress-induced pathway response

**Figure 3:**
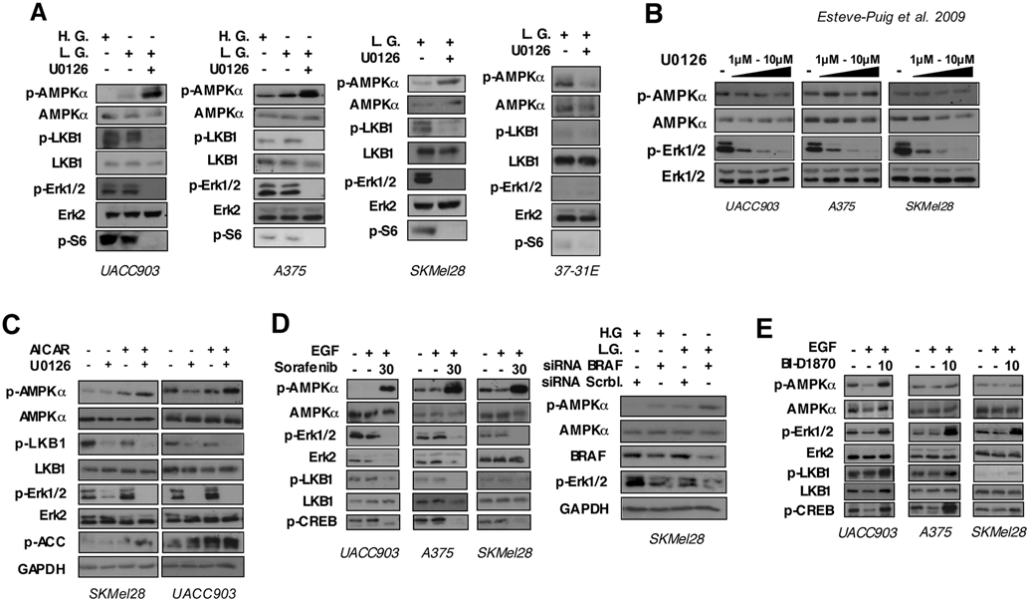
Inhibition of oncogenic BRAF^V600E^ signaling restores the limited response to metabolic stress of BRAF mutant melanoma cell lines. (A) BRAF mutant melanoma cells have a limited response to energy withdrawal that is restored by U0126 treatment. Fifty micrograms of total lysates from UACC903, A375, SKMel28 and 37-31E melanoma cells grown in serum free high glucose medium (H.G.), serum free low glucose medium (L.G.) or serum free low glucose medium (L.G.) plus 10 µM of U0126 for 4 hours were separated by SDS-PAGE. Western-Blot shows the activation status of proteins in the RAS and LKB1-AMPK-mTOR pathways. (B) U0126 inhibitor treatment does not activate AMPK. UACC903 A375 and SKMel28 melanoma cells were grown in high glucose medium with serum in the absence or presence of 1 µM, 5 µM or 10 µM of U0126. Total protein lysates were subjected to SDS-PAGE. Western-blot shows the phosphorylation state of AMPKα in the presence of different concentrations of U0126. (C) Inhibition of BRAF signaling increases cell response to AICAR. UACC903 and SKMel28 cells were grown in complete medium; cells were treated with AICAR (1 mM) for 4 h in the presence or absence of U0126 (10 µM) inhibitor. p-LKB1^Ser428^, p-AMPK^Thr172^, p-Erk1/2^Thr202/Tyr204^, p-ACC^Ser79^ levels were checked by western blot. (D) Sorafenib treatment and siRNA BRAF knockdown restores the metabolic stress pathway in BRAF mutant melanoma cells. In the left panel, UACC903, A375 and SKMel28 melanoma cells were grown in low glucose serum free medium+/−EGF (50 ng/ml) for 4 h in the presence or absence of U0126 (10 µM) Western blots show the levels of p-AMPKα^T172^, p-LKB1^Ser431^ p-Erk1/2^Thr202/Tyr204^ and pCREB^Ser133^ proteins under the different conditions. In the right panel SKMel28 cells were transfected with either a scramble siRNA or BRAF siRNA. 72 hours after transfection, cells were starved either in high glucose (H.G.) or low glucose (L.G.) medium for six hours. Western-blots show the levels of p-AMPKα^T172^, p-Erk1/2^Thr202/Tyr204^ and BRAF proteins. (E) p90^Rsk^ inhibitor BI-D1870 (10 µM), does not restore the metabolic stress pathway. UACC903, A375 and SKMel28 melanoma cells were grown in low glucose serum free medium+/−EGF (50 ng/ml) for 4 h in the presence or absence of BI-D1870 (10 µM). Western blots show the levels of the indicated proteins under the different conditions.

Melanoma cells are especially resistant to different types of stress. LKB1 is the AMPKα upstream kinase that becomes phosphorylated in response to metabolic stress controlling protein synthesis through mTOR pathway. Considering the LKB1^Ser428^ phosphorylation as a read out of the RAS and LKB1 pathways interaction, the constitutive phosphorylation of LKB1^Ser428^ in BRAF mutant cells suggested a possible interplay between LKB1-AMPK pathway and BRAF oncogenic signaling. Therefore, we investigated the LKB1-AMPK pathway activation in three different BRAF^V600E^ mutant melanoma cell lines under low energy conditions and the contribution of BRAF^V600E^ signaling to the energy sensor pathway. To test this hypothesis we starved BRAF mutant melanoma cells under serum free and low glucose conditions in the presence or absence of the Mek1/2 inhibitor U0126, and investigated the activation of LKB1-AMPK-mTOR pathway. UACC903, SKMel28 and A375 cells showed a very limited response to energy withdrawal as measured by the induction of phospho-AMPKα (Fig. 3A). Under these conditions all cells retained considerable mTOR activity as indicated by the phosphorylation levels of ribosomal protein S6 (Fig. 3A). However, the addition of U0126 (10 µM) recovered AMPKα pathway activation in response to low energy conditions resulting in the complete abrogation of mTOR activity as indicated by phospho-S6 ribosomal protein (Fig. 3A). In contrast, 37-31E melanoma cells harboring wild type BRAF did not show this effect by the addition of U0126 under low energy conditions (Fig. 3A). Interestingly, the re-activation of the AMPKα pathway in BRAF mutant cells correlated with the total inactivation of Erk1/2 and the unphosphorylated LKB1^S428^ (Fig. 3A). Importantly, the addition of U0126 up to 10 µM in serum free high glucose conditions did not induce the activation of AMPKα [36] (Fig. 3B). To confirm the reconnection of the AMPK pathway after inhibition of oncogenic BRAF signaling we used AICAR (5-Aminoimidazole-4-carboxyamide ribonucleoside) instead of low glucose in order to stimulate the activation of the AMPK pathway. As expected, the addition of AICAR, which increases AMPK phosphorylation levels by a mechanism that appears to be due to the inhibition of AMPK dephosphorylation [37], [38], [39], slightly increased the p-AMPK levels in serum starvation. The addition of U0126 inhibitor resulted in a clear increment the p-AMPKα levels (Fig. 3C). Since this effect was observed in BRAF^V600E^ mutant cells, we repeated the experiments using the BRAF inhibitor sorafenib. Notably, inhibition of BRAF^V600E^ signaling with sorafenib recovered the activation of AMPK pathway in response to metabolic stress. Interestingly, sorafenib treatment under low glucose condition reduced Erk2 protein levels by a currently unknown mechanism (Fig. 3D). Importantly, experiments knocking–down BRAF^V600E^ performed in serum free and low glucose medium also resulted in an increased of p-AMPKα levels (Fig 3D).

In addition, we tested whether p90^RSK^ signaling was mediating the observed effect using the p90RSK specific inhibitor BI-D1870. Experiments were done in the presence of EGF to assured the activation of RAS pathway. Treatment of cells with the p90^RSK^ inhibitor BI-D1830 did not recover the cells response to low energy conditions as indicated by the p-AMPK levels (Fig. 3E) suggesting, that the oncogenic BRAF-mediated LKB1^Ser428^ phosphorylation was not sufficient to account for the observed response. As previously shown, BI-D1870 treatment induced the Erk1/2 and CREB^Ser133^ phosphorylation mediated by the suggested p90^RSK^ negative-feedback loop that regulates Erk1/2 [20].

Altogether these data show evidences that indicate that melanoma cells harboring oncogenic BRAF have a diminished response to metabolic stress. Importantly, the inhibition of the oncogenic BRAF signaling, that connects RAS pathway to LKB1, restored the AMPK-mediated energy stress sensor pathway. However the results also indicate that the inhibition of the LKB1^Ser428^ phosphorylation is not enough to recover the pathway LKB1-AMPK-mTOR response, suggesting the existence of additional mechanisms.

### Growth factor treatment and oncogenic BRAF^V600E^ induce LKB1-AMPK disassembly

**Figure 4:**
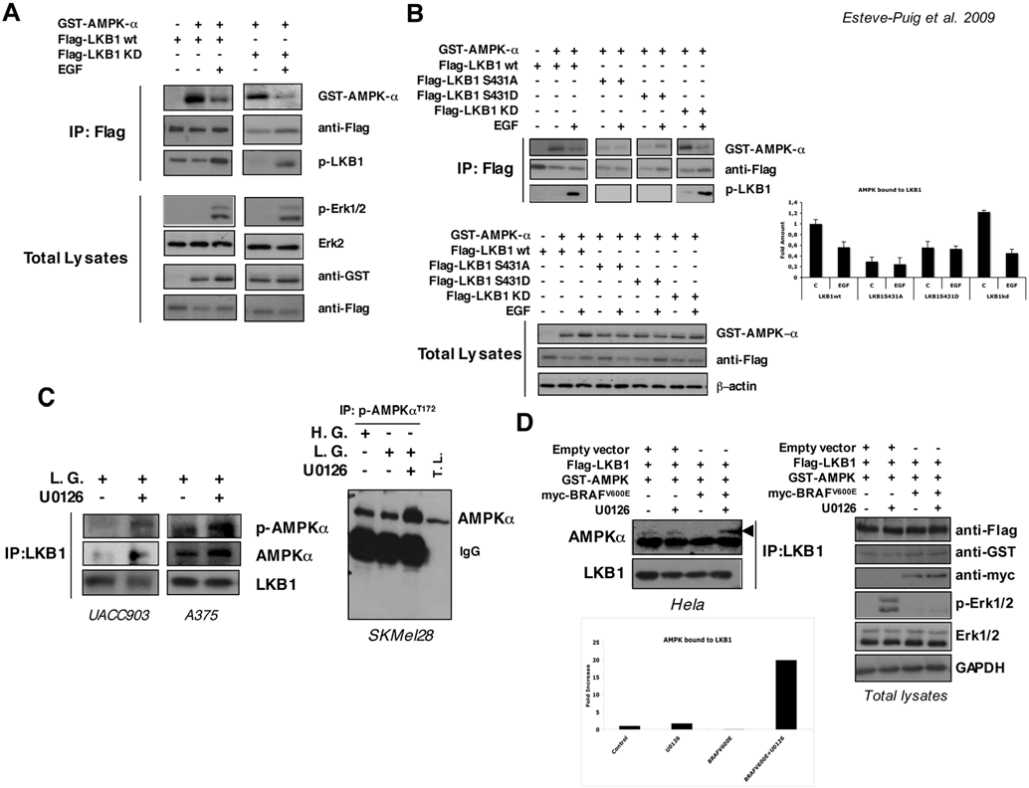
Growth factor treatment and BRAF^V600E^ promotes LKB1-AMPKα disassembly. (A) 293T cells were transiently transfected for 48 h with Flag-LKB1, Flag-LKB1KD (kinase dead) and GST-AMPKα as indicated. Then, cells were treated with 100 ng/ml of EGF for 10 min. Immunocomplexes pulled down with an anti-Flag-resin were separated by SDS-PAGE and proteins present in the complexes were analyzed by western blot. Total lysates show the transfection controls and the response to growth factor treatment. (B) 293T cells were transiently transfected with the constructs indicated. Then, cells were serum starved for 2 h and treated with 100 ng/ml of EGF for 10 min and protein complexes were immunoprecipitated with anti-Flag-resin. Protein complexes were separated by SDS-PAGE. Levels of GST-AMPKα, Flag-LKB1 constructs and the phosphorylation state of LKB1^Ser431^ in the complexes are shown. Quantification of the amount of GST-AMPKα normalized to the Flag-LKB1 immunoprecipitated is represented in the graph. Total lysates are shown for control transfection of the different samples. (C) Endogenous LKB1 from UACC903 and A375 melanoma cells growing in low glucose medium (L.G.) with or without 10 µM U0126 was immunoprecipitated. Western-blot from the immunoprecipitated samples was probed against LKB1, AMPKα and p-AMPKα^T172^ antibodies. On the right, total lysates from SKMel28 melanoma cells growing in complete medium (High Glucose, H.G.) low glucose medium (L.G.) in the presence or absence of 10 µM U0126 were subjected to immunoprecipitation with the anti-p-AMPKα^T172^. Samples were separated by SDS-PAGE. Total lysates (T.L.) from low glucose plus U0126 treated cells are showed as a control. Western-Blot of the immunoprecipitated samples was performed against total AMPKα antibody. (D) Hela cells were transfected with Flag-LKB1, GST-AMPKα and myc-BRAF^V600E^ or and empty vector as indicated. Flag-LKB1 was immunoprecipitated and western-blots from immunoprecipitated samples were probed against the indicated antibodies. Graph shows the quantification of the AMPK bound to LKB1.

The LKB1 tumor suppressor kinase activity is not related to its phosphorylation state [14]. Thus, its contribution to the different biological processes is likely to be mediated by its interaction with other proteins and/or its cellular localization. LKB1 controls protein synthesis and cell growth through the AMPK-TSC1/TSC2 cascade [15], [16], [17]. Mitogenic responses coordinate simultaneously cell growth with cell division. Since the activation of AMPK-TSC1/TSC2 pathway by LKB1 controls energy metabolism and protein synthesis, we examined whether the growth factor treatment leads to LKB1-AMPK dissociation, providing a mechanism that would assure cell growth upon a mitogenic stimulus and resistance to energy stress conditions. To investigate the underlying mechanism, we transfected 293T cells with Flag-tagged LKB1 and GST-AMPKα and then treated the cells with or without EGF in order to activate RAS pathway. After immunoprecipitation of the Flag-LKB1 complexes we checked for the presence of GST-AMPKα in the immunocomplexes. The data indicated that some fraction of AMPKα is constitutively bound to LKB1. Interestingly, treatment of cells with the growth factor induced dissociation of the LKB1-AMPKα complexes (Fig. 4A). Moreover, this effect was totally independent of LKB1 kinase activity since Flag-tagged kinase dead LKB1 (LKB1^KD^) reproduced exactly the same result. Since growth factor stimulation promoted LKB1-AMPKα disassembly and this effect correlated with the phosphorylation of LKB1^Ser428^ upon growth factor stimulation, we examined the role of the Ser428 (Ser431 in mouse) residue on this effect. We transfected LKB1^WT^ wild type, LKB1^S431A^ mutant, LKB1^S431D^ phospho-mimetic mutant and the LKB1^KD^ constructs together with GST-AMPKα in 293T cells and repeated the previous experiment. Again when LKB1^WT^ and LKB1^KD^ were transfected the EGF treatment promoted the disassembly of the LKB1-AMPKα complex. However, GST-AMPKα did not form a complex with either the LKB1^S431A^ or LKB1^S431D^ mutants, suggesting that, in response to growth factors, the Ser428 residue would be involved in the binding or stability of the LKB1-AMPKα complexes (Fig. 4B).

The above data suggested that the limited response to metabolic stress of BRAF mutant melanoma cells could be caused by the dissociation of LKB1-AMPKα complexes. Thus, the inhibition of BRAF signaling by U0126 inhibitor would permit the reconnection of the pathway. To confirm that, we performed an immunoprecipitation of the endogenous LKB1 in the BRAF mutant melanoma cells and examined the AMPKα association under low energy conditions with or without U0126 inhibitor. UACC903 and A375 melanoma cells showed an increase in the number of AMPKα molecules associated to LKB1 when BRAF signaling pathway was blocked (Fig. 4C). This re-assembly was also associated with an increase in AMPKα^T172^ phosphorylation levels. Similar results were obtained when endogenous p-AMPKα^T172^ was immunoprecipitated from SKMel28 melanoma cells under same conditions, confirming the suggested mechanism (Fig. 4C). Additionally, we reconstitute the system in Hela cells that do not express endogenous LKB1. Hela cells were transfected with Flag-LKB1 and GST-AMPKα in the presence or absence of oncogenic myc-BRAF^V600E^ under low glucose conditions. The expression of oncogenic BRAF^V600E^ induced the complex dissociation that was totally rescued by the addition of U0126 inhibitor (Fig. 4D).

These data showed evidences that support the growth factor treatment and RAS pathway activation mediated disassembly of the LKB1-AMPK complexes. Importantly the inhibition of the RAS pathway in cells harboring BRAF^V600E^ mutation restores the LKB1-AMPKα pathway by permitting the re-association of the LKB1-AMPKα complexes. Furthermore, these data also suggested that although growth factor stimulation induces LKB1^Ser428^ phosphorylation, additional mechanisms should be involved promoting the RAS pathway-dependent LKB1-AMPK disassembly.

### Restoration of the LKB1-AMPKα pathway in BRAF^V600E^ melanoma cells under energy stress conditions induces apoptosis in coordination with Bad, Bim and Mcl-1

**Figure 5:**
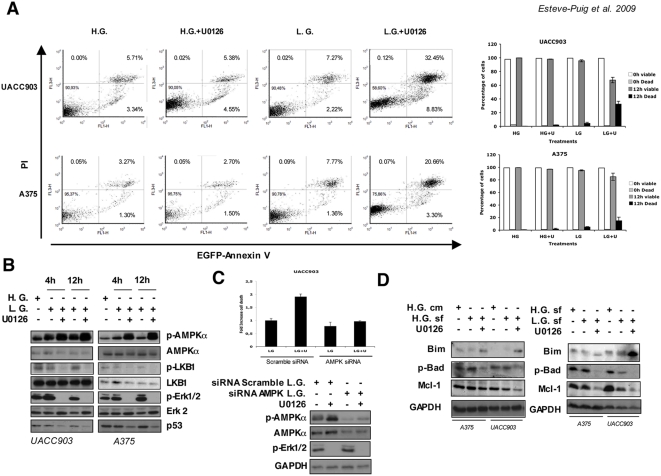
Restoration of the LKB1-AMPKα pathway in BRAF^V600E^ melanoma cells induces apoptosis under energy stress conditions. (A) UACC903, A375 and SKMel28 human melanoma cells were grown in complete medium (H.G.), or low glucose medium (L.G.) with or without 10 µM of the Mek1/2 specific inhibitor U0126 for 12 h. Then, Annexin V and PI (propidium iodide) positive cells were analyzed by flow cytometry. Histograms show the result from FACS analysis. Graphs on the right show the percentage of viable and dead cells in a parallel experiment under the same conditions determined by nuclear staining exclusion (Guava-ViaCount). (B) Time course at 4 and 12 hours showing the LKB1-AMPKα pathway status under the same conditions. UACC903, A375 and SKMel28 human melanoma cells were grown in complete medium (high glucose H.G.), low glucose medium (L.G.) with or without 10 µM of the Mek1/2 specific inhibitor U0126 for the times indicated. Fifty micrograms of total protein lysates were separated by SDS-PAGE and same membranes were blotted against the indicated antibodies. All experiments were done at least three times. Representative experiments are shown. (C) UACC903 cells were transfected either with a scramble siRNA or with equimolar amounts of AMPKα1 and AMPKα2 siRNAs for a total concentration of 100 nM. 72 hours after transfection cells were starved in low glucose medium for 6 hours in the presence or absence of 10 µM of U0126. Dead cells were quantified by nuclear staining exclusion (Guava-ViaCount). Western-blots show the levels of p-AMPKα^T172^, AMPKα and p-Erk1/2^Thr202/Tyr204^ under the different conditions. (D) UACC903 and A375 melanoma cells were grown in complete medium (H.G. cm), serum free high glucose medium (H.G. sf), serum free low glucose medium (L.G.), serum free complete medium plus U0126 10 µM (H.G.+U0126) and low glucose serum free medium plus U0126 10 µM (L.G.+U0126) for 12 hours. The levels of Bim, phospho-Bad and Mcl-1 are showed under the different experimental conditions.

Next, investigated the cell survival response of BRAF^V600E^ mutant melanoma cell lines with a restored LKB1-AMPKα pathway under stress energy conditions. According to the accepted mechanism, elevation of intracellular AMP levels will activate LKB1 that in turn activates AMPK and regulates apoptosis in response to energy stress [16]. Additionally, it has been shown that blocking BRAF signaling in BRAF^V600E^ mutant cell lines for long periods of time (24–48 hours) promotes apoptosis through the regulation of BH3-family proteins [40], [41]. We subjected UACC903 and A375 melanoma cells to metabolic stress conditions for a maximum of 12 hours in the presence or absence of the Mek1/2 inhibitor. Then, we measured cell viability and apoptosis by nuclear staining exclusion (Guava ViaCount) and Annexin V and propidium iodide (PI) double staining. UACC903 and A375 melanoma cell lines showed some spontaneous apoptosis under normal growing conditions: 5.71% and 3.27% respectively. The addition of Mek1/2 inhibitor for 12 hours in high glucose medium did not promote any increment in the apoptosis rate (UACC903 5.38% and A375 2.70%). Glucose starvation for 12 hours resulted in slight increase in the number of double positive Annexin V-PI cells respect normal growing conditions: 1.27 fold for UACC903 cells and 2.35 fold for A375 cells. However, the restoration of the LKB1-AMPKα pathway by inhibition of Mek1/2 under low glucose conditions resulted in a considerable number of apoptotic cells (5.7 and 6.4 fold increase respectively); (Fig 5A). Similar results were observed when viable cells were analyzed by PI exclusion (Fig. 5A). The results were correlated with the molecular status of the pathways implicated in a time course fashion. As shown before, the inhibition of Erk1/2 phosphorylation resulted in the decrease of phopho-LKB1^Ser428^ levels and the restoration of the LKB1-AMPKα pathway. In turn, the LKB1-AMPKα pathway was able to sense the low energy conditions as soon as 4 hours after glucose starvation, indicated by the levels of p-AMPKα^T172^ (Fig. 5B). Interestingly, the increase in the number of apoptotic cells did not correlate with p53 stabilization. On the contrary, p53 levels were down-regulated under these conditions in all cell lines, suggesting the participation of BRAF signaling in the stabilization of p53 and a p53-independent apoptotic mechanism (Fig. 5B).

In order to establish a causal link among the inhibition of BRAF signaling, the AMPK pathway re-activation, and the increased number of dead cells, we knocked-down AMPKα in UACC903 melanoma cells and investigated the response under low glucose conditions to the inhibition of the oncogenic BRAF signaling. As showed in figure 5C, blocking BRAF signal under low energy conditions in control cells resulted in elevated levels of p-AMPKα^T172^ together with an increment in the number of dead cells. However, AMPKα knock–down cells did not show any increase in the number of dead cells under similar conditions.

As mentioned previously, BRAF suppresses apoptosis, targeting the BH3-family of proteins in BRAF^V600E^ mutant cells [40]. In high glucose medium the addition of the U0126 inhibitor for 12 hours caused a small decrease of p-Bad and the Bcl-2 family member Mcl-1 protein levels together with a slight increase in the amount of Bim_EL_. Interestingly, when the LKB1-AMPKα pathway was restored under low glucose conditions, the increased number of dead cells correlated with a stronger biochemical response including the complete de-phosphorylation of Bad, the stabilization of the non-phosphorylated Bim_EL_ isoform and the drastic down-regulation of Mcl-1 (Fig. 5D).

Our results indicated that in a BRAF mutant context, the reactivation of LKB1-AMPK-mTOR pathway under low energy conditions together with the inhibition of oncogenic BRAF signaling for short periods of time, promoted a pronounced apoptosis response through the de-phosphorylation of Bad, stabilization of Bim_EL_ and the down-regulation of Mcl-1.

## Discussion

The understanding of the molecular and biochemical mechanisms contributing to melanoma development and progression is critical for therapeutical intervention. The UV-induced HGF mouse melanoma model recapitulates chronologically and histopathologically all the stages of human melanoma [6], [7]. We investigated the potential interplay among the HGF RTK signaling, the RAS-Erk1/2 and the PI3K-AKT pathways using the HGF mouse model. In neoplasic melanoma cells isolated from spontaneous tumors raised in the mouse model, we identified LKB1 as one of the molecules responsive to HGF triggering. As previously described for EGF [18], we show that HGF and several other growth factors induce the phosphorylation of LKB1^Ser431^ through the Ras-Erk1/2-p90^RSK^ pathway. Interestingly, this residue appears to be constitutively phosphorylated in human melanoma cells harboring BRAF^V600E^ activating mutation as an indicator of the connection between RAS pathway and LKB1. The role of LKB1 in response to growth factors, and its connection to the RAS pathway, is mostly unknown. Our results show that melanoma cells harboring the BRAF^V600E^ oncogenic mutation have a very limited response to metabolic stress. Interestingly, the inhibition of the BRAF signaling restores the ability of the cells to sense the low energy conditions. Notably, growth factor treatment and oncogenic BRAF^V600E^ leads to the uncoupling of LKB1-AMPKα complexes, suggesting a mechanism that disconnects the energy sensor pathway, which is involved in controlling cell growth through the mTOR pathway in response to low energy conditions. Furthermore, inhibition of BRAF oncogenic signaling promotes the association of the LKB1-AMPKα complexes and results in an increase of apoptosis in response to metabolic stress.

Our screening for the discovery of novel molecules involved in HGF signaling in melanoma cells allowed us to identify by DIGE analysis proteins that were directly modified in response to c-Met activation by HGF (data not shown). Previously, it has been described that EGF and forskolin promote the phosphorylation of LKB1^Ser428^ in a p90^RSK^ and PKA dependent manner respectively [18], [19]. Our results showed that LKB1^Ser431^ phosphorylation occurs in response to HGF and other different growth factors in a RAS-Erk1/2-p90^RSK^ dependent manner as initially suggested by previous investigations [18], [19]. Interestingly, and according to siRNA knock down experiments (data not shown), cells expressing less than five percent of the LKB1 pool still respond to this stimulus, suggesting that LKB1 would have a relevant role in the mitogenic response to growth factors. Virtually all cancers have aberrant signaling of receptor tyrosine kinases (RTKs), growth factors autocrine loops or activating mutations in the RAS pathway (RAS activating mutations or BRAF^V600E^ mutation). We therefore hypothesized that LKB1 would be mediating some of the effects of the RAS and BRAF oncogenes. In agreement with this, LKB1^Ser428^ appears to be constitutively phosphorylated in human melanoma cell lines harboring BRAF^V600E^ activating mutations as an indicator of the interplay between RAS pathway and LKB1. Furthermore, LKB1^Ser431^ (Ser428 in human) tends to be phosphorylated in mouse tumor samples harboring deregulated tyrosine kinase activities or increased mitogenic signaling, suggesting the direct participation of LKB1 in tumor biology.

LKB1 activity is controlled through its interaction with the STE20-related adaptor (STRAD) and Mo25 [13], [14]. LKB1 can be phosphorylated at eight or more different residues, where the modifications at these amino acids have no effect on LKB1 kinase activity [8]. In the last five years a number of publications have reported several critical roles for LKB1 in different biological processes such as: energy metabolism [11], [16], cell polarity and division [12], [11] and transcriptional regulation [10]. However, most of these studies rely on the presence or absence of LKB1 in these processes. Our data show that growth factor treatment induces both, LKB1^Ser428^ phosphorylation and the dissociation of LKB1-AMPKα complexes. The participation of this residue in growth inhibition, cell polarity and the activation of the LKB1 downstream kinases (AMPK, BRSK1/2) have been controversial [8], [21], [23], [25], [26]. Indeed, our results regarding the participation of this residue in the growth factor-mediated dissociation of the LKB1-AMPKα complexes are not conclusive and more research is needed in order to elucidate the complete role of this residue. Moreover, we can no exclude the participation of additional residues or proteins in the process.

**Figure 6:**
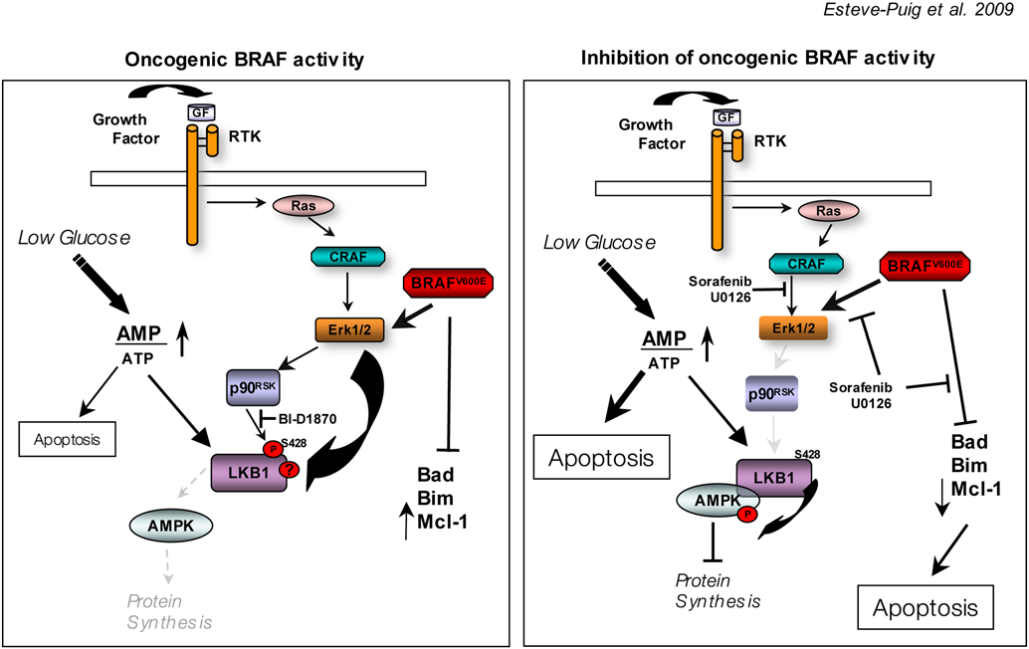
A model of the metabolic stress response regulation by oncogenic BRAF in melanoma cells. Resistance to stress conditions is essential for melanoma cells survival. We propose that oncogenic BRAF^V600E^ signaling (left panel) protects to apoptosis by regulating BH3-family members and confers resistance to low energy conditions promoting the uncoupling of LKB1 and AMPK through Erk1/2 and p90^Rsk^. Under this condition BRAF mutant cells have a limited response to low energy conditions. On the right panel the inhibition of BRAF signaling allows the formation of the LKB1-AMPK complexes restoring the energy stress pathway and promoting the down-regulation of anti-apoptotic proteins such as Mcl1. The activation of AMPK by metabolic stress conditions and the inhibition of BRAF signaling would have synergistic effects promoting apoptosis.

LKB1 is the upstream kinase of AMPKα and is linked to mTOR through the AMPK-TSC1/TSC2 cascade [15], [16], [17] controlling cell growth under energy stress conditions. One possible interpretation of our results would be that the dissociation of the LKB1-AMPKα complex would provide a mechanism to avoid interruption of protein synthesis through this pathway while cells are responding to a mitogenic stimulus. In this matter, the activation of RAS-Erk1/2 pathway would engage biochemical mechanisms to coordinate cell growth and division to assure cell proliferation. Importantly, the deregulation of the AMPK-mTOR axis by the dissociation of the LKB1-AMPKα complex in cells harboring RAS pathway activating mutations could represent an advantage for proliferation and a significant resistance increased to metabolic stress conditions. Notably, BRAF^V600E^ mutant melanoma cell lines showed a limited sensitivity in response to low energy conditions. Treatment of cells with the Mek1/2 inhibitor U0126 allowed the formation of endogenous LKB1-AMPKα complexes and restored the energy sensor pathway in response to low energy conditions, supporting the proposed mechanism (Fig. 6). Mek1/2 inhibitors (U0126, PD98059) have been reported to activate AMPK at 20 µM concentration, but not at 5–10 µM [36]. These experiments were done in glucose free medium and in the presence of growth factors with high levels of phospho-Erk1/2. According to our data the inhibition of the Erk1/2 pathway under low energy conditions would allow the re-association of LKB1-AMPKα, which in turn would result in an increase the levels of p-AMPKα. Furthermore, our data showed that in high glucose medium the addition of U0126 (10 µM) or sorafenib (data not shown) had no effect on AMPKα phosphorylation in all BRAF mutant cell lines (Fig 5A). Up-to-date the connection between RAS pathway and LKB1 has been limited to the phosphorylation of LKB1^Ser428^ residue through p90^RSK^. However, our results indicate that this modification is not enough to mediate the observed effect, suggesting the existence of additional biochemical mechanisms mediated by Erk1/2 that will account for the LKB1-AMPKα dissociation mediated by the RAS pathway (Fig. 6).

The activation of AMPKα by LKB1 under energy stress, stimulates glucose uptake and fatty acid oxidation to increase ATP production, inhibits protein synthesis and protects cells from undergoing apoptosis [8]. Surprisingly, the inhibition of Erk1/2 pathway in BRAF mutant melanoma cell lines subjected to metabolic stress resulted in an increase in the number of dead cells (Fig 6). Interestingly, our experiments knocking-down AMPKα suggests a causal link among the inhibition of oncogenic BRAF signaling, the reconstitution of the energy sensor pathway and the resulting cell death. In agreement with the interplay between the oncogenic signaling and AMPK is the recent finding where the activation of AMPK pathway by administration of metformin, phenformin or A-769662 to PTEN(+/−) mice significantly delayed tumor onset, demonstrating that LKB1 is required for activators of AMPK to inhibit mTORC1 signaling as well as cell growth in PTEN-deficient cells [42]. Interestingly, the increased apoptosis rate did not correlate with the stabilization of p53, indicating that the mechanism was apparently p53-independent and that BRAF oncogenic signaling was participating in the stabilization of p53. In fact, it is known that under genotoxic-stress conditions, Erk1/2 signaling mediates p53 stabilization [43], [44]. Furthermore, the AMPK-induced p53 activation has been reported to promote cell survival in response to glucose deprivation in MEFs [45], while our data in BRAF mutated melanoma cells clearly showed an increase in apoptosis.

Recent publications have shown that oncogenic BRAF can suppress apoptosis through targeting BH3-only proteins Bad and Bim [40], [41]. Our results indicate that the inhibition of oncogenic BRAF signaling at 12 h promotes a slight de-phosphorylation of Bad and the stabilization of Bim_EL_, most likely, by inhibiting its Erk1/2-dependent phosphorylation and proteasome-mediated degradation [46]. The reactivation of the LKB1-AMPK-mTOR pathway under low energy conditions by the inhibition of BRAF signaling led to a more pronounced effect that included a drastic down-regulation of Mcl-1 (Fig 6). Interestingly, Mcl-1 has been shown to be an important melanoma anti-apoptotic protein [47].

In conclusion, in this report we show that activation of RAS pathway by growth factors and oncogenic BRAF^V600E^ results in the dissociation of LKB1-AMPKα. These results, permit us to speculate that under normal growth conditions, this biochemical mechanism, through the activation of RAS pathway, could be involved in the coordination of two important processes in cell proliferation: cell growth and cell division. Interestingly, BRAF^V600E^ mutant melanoma cells have minimal response to energy stressed conditions due to the constitutive dissociation of the LKB1-AMPKα complexes. However, under metabolic stress conditions the inactivation of BRAF oncogenic signaling restores the LKB1-AMPKα-mTOR pathway-promoting apoptosis in collaboration with BH3-only proteins and Mcl-1 (Fig 6). Importantly, this mechanism reveals a new level for therapeutical intervention triggering apoptosis of tumor cells. This might be particularly relevant in tumors harboring a deregulated RAF-Erk1/2 pathway that survive in energy stress conditions.

## Materials and Methods

### Cell lines

37-31E and 37-31T cells have been described previously [33]. The 293T, Hela, SKMel28, MeWo, A375 and B16F1 cell lines were from ATCC. The 293T cells and Hela cells were maintained in DMEM (Gibco) with 10% FBS and penicillin/streptomycin, while the 37-31E and 37-31T cells were supplemented with EGF (5 ng/ml) (Invitrogen) and Insulin (4 µg/ml) (Invitrogen). The human melanoma cell lines SKMel28 and were grown in MEM (ATCC) supplemented with 10% FBS plus penicillin/streptomycin (Gibco). UACC903 were a gift from J. Trent (P. Pollock), Tgen Phoenix, Arizona. All cell lines were growth under 37°C and 5% CO_2_ conditions. For low glucose conditions cells were put in DMEM (Gibco) or MEM (Gibco) low glucose medium plus penicillin/streptomycin for at least 4 h.

### Phospho-protein isolation

37-31E cells were serum starved for two hours and then triggered with 40 ng/ml of HGF (R&D) for 10 minutes in the presence or absence of 0.2 µM of the c-Met specific inhibitor PHA (Sugen-Pfizer). Cells were then lysed according to the phospho-protein purification kit (Qiagen Inc.), and phospho-proteins purified according to manufacturer instructions.

### Plasmids

pCMV5-Flag-LKB1^WT^ wild type, pCMV5-Flag-LKB1^KD^ kinase dead, pCMV5-Flag-LKB1^S431A^ mutant and pCMV5-Flag-LKB1^S431D^ mutant were obtained from Dario Alessi (University of Dundee). pEBG-2t-GST-AMPKα was a kind gift from José Miguel Lizcano and José Manuel López Blanco (Autonomous University of Barcelona). pLPCX-myc-BRAF^V600E^, was subcloned from pEB- myc-BRAF^V600E^ (obtained from Richard Marais, ICRF).

### Cell transfection

293T cells were seeded at 60% confluence the day before transfection. Cells were transiently transfected with Lipofectamine reagent (Invitrogen Inc.) following the manufacturer's protocol. Cells were treated and lysed 36–48 h after transfection.

### siRNA transfection experiments

Scramble siRNA, human BRAF siRNA On target-smartpool, and human siRNA AMPKα1 and AMPKα2 On-target-smartpools were purchased from Dharmacon. 100 nM siRNA was transfected into cells using Lipofectamine 2000 (Invitrogen Inc.) following manufacturer protocol. Experiments were performed 72 hours after transfection.

### Reagents and Western Blot analysis

PHA c-Met specific inhibitor (Pfizer) was diluted in DMSO and used at the concentrations indicated. Mek1/2 inhibitor U0126 and PI3K inhibitor LY294002 (Cell Signaling) were used at 10 µM concentration. P90RSK inhibitor BI-D1870 was purchased from MSI/WTB University of Dundee and used at 10 µM. Cells were treated with the inhibitors for 2 hours under serum starvation and then treated with HGF (40 ng/ml) for 10 min. Five µg of phospho-proteins or 50 µg of total protein lysates were separated by SDS-PAGE and transferred to a PVDF membrane (Millipore). The membranes were blocked in 5% milk (Santa Cruz) and blotted against different primary antibodies. ERK2 and LKB1 were from Santa Cruz. Anti-DYKDDDDK (Flag), phospho-Erk1/2 (Thr202/Tyr204), Erk1/2, phospho-ACC (Ser79), p-90^RSK^ (Thr359/Ser363), AMPKα, p-AMPK (Thr172), phospho-S6 ribosomal protein (Ser235/236); phosho-Bad (Ser112), anti-Bad and Bim were purchased from Cell Signaling. Additionally p-Bad (Ser112) was purchased from Genscript Co., Anti-GST polyclonal antibody and anti-Flag was purchased from Sigma-Aldrich and Genscript Co. and GAPDH was purchased from Trevigen. Mcl-1 antibody was from DAKO. Anti-Flag resin was obtained from Sigma-Aldrich and glutathione-resin was purchased from Amersham and Genscript Co. Membranes were developed using horseradish linked secondary antibodies (GE Healthcare) and ECL (GE Healthcare).

### Immunoprecipitations

36–48 h after transfection cells were treated as needed and lysed in RIPA Buffer containing a protease cocktail II inhibitor (Sigma-Aldrich). 800–1000 µg of total protein was subjected to immunoprecipitation with 30 µl Flag-resin or 30 µl of Glutathion-resin. Then, samples were washed three times with RIPA buffer and SDS-loading sample buffer was added to the samples. Samples were separated by SDS-PAGE.

### Cell viability and apoptosis assays

Cell viability and dead cells were counted by using Guava ViaCount reagent (Gevara Technologies) cell counter (ViaCount). Apoptosis was measured using the Annexin V-EGFP apoptosis detection kit (Genscript corporation) following the manufacturer's protocol. Positive cells for Annexin V-EGFP and propidium iodide staining were analyzed and quantified by flow cytometry (FACScalibur).

### Image analysis

Bands from tumor samples were quantified using NIH1.6 Image software. Normalization of p-proteins was performed against the normalized amount of the total phosphorylated protein. Other proteins were normalized against GAPDH.
